# Relationship between Brain Natriuretic Peptide and Thromboembolic Events in Elderly Patients with Nonvalvular Atrial Fibrillation

**DOI:** 10.1155/2024/5594637

**Published:** 2024-01-17

**Authors:** Hongxia Wang, Jiajun Huang, Wenxi Gu, Xiaojiao Hao, Guiru Li, Yumin Yuan, Yingmin Lu

**Affiliations:** Department of Cardiology, Chongming Hospital Affiliated to Shanghai University of Medicine and Health Sciences, Shanghai 200000, China

## Abstract

**Objective:**

To investigate the relationship between brain natriuretic peptide (BNP) and thromboembolic events in elderly patients with nonvalvular atrial fibrillation (NVAF).

**Methods:**

This is a prospective cohort study, and based on the inclusion and exclusion criteria, 180 elderly patients with NVAF were included. The patients received follow-up appointments in the clinic or by telephone every 6 months after the beginning of the study. The primary follow-up endpoints were thromboembolic and atherosclerotic events, including ischaemic stroke, myocardial infarction, and systemic embolism. The secondary endpoints were adverse events, including cardiovascular death, all-cause death, and hospitalisation for heart failure. Patients were divided into three groups according to their BNP level at admission: group A (BNP ≤334.5 pg/mL), group B (BNP = 334.5–1,288 pg/mL), and group C (BNP ≥1,288 pg/mL).

**Results:**

A total of 180 patients were enrolled in this study, with 50 patients in group A, 68 in group B, and 62 in group C. Compared with groups A and B, group C had a higher CHA2DS2-VASc score (*Z* = 15.142; *P*=0.001) and a lower ejection fraction (EF) value (*Z* = 119.893; *P*=0.001). The left atrium (LA) and left ventricular end-diastolic diameter (LVEDD) were larger (*Z* = 105.031; *P*=0.001 and *Z* = 74.430; *P*=0.001), respectively, suggesting that patients with significantly increased BNP had a higher risk of thromboembolism and atherosclerosis, lower EF, larger LA and LVEDD, and worse cardiac function. After 1 year of follow-up, the incidence of primary endpoint events (*χ*^2^ = 9.556; *P*=0.008) and secondary endpoint events (*χ*^2^ = 59.485; *P*=0.001) in group C were higher than those in groups A and B.

**Conclusion:**

Higher BNP levels may be an independent risk factor for thromboembolic and atherosclerotic events in elderly patients with NVAF. The higher the BNP level, the greater the risk of thromboembolic and atherosclerotic events.

## 1. Introduction

In Chinese population, the prevalence of atrial fibrillation (AF) is approximately 2% in those aged >45 years, reaching 5% in those over 75 years [1]. The incidence of AF is expected to triple in the coming decades. Nonvalvular AF (NVAF) is the most common clinical arrhythmia, with an estimated prevalence of 1.5%–2.0% worldwide [2]. Atrial fibrillation has gained increasing attention in recent years because of its severe complications, which include stroke, heart failure (HF), and sudden death, and it has a high fatality rate [3]. Studies have demonstrated that the risk of ischaemic stroke in patients with NVAF is approximately five times higher than that in patients without it, and the disability rate of stroke is also two times higher [4]. Heart failure occurs in 20%–30% of patients with AF, and AF also occurs in more than half of the patients with HF. Many epidemiological investigations have also identified HF as a key risk factor for AF onset, and its incidence increases as the severity of HF increases [5, 6].

Brain natriuretic peptide (BNP) and N-terminal probrain natriuretic peptide (NT-proBNP) are considered the most valuable and reliable biomarkers for the diagnosis of heart disease and cardiac insufficiency and are used to assess the severity of the former, guiding the relevant treatment strategy and evaluating the prognosis [7, 8]. Both are routine examination indicators for patient admission. Brain natriuretic peptide is mainly produced by cardiomyocytes in response to increased end-diastolic pressure and/or volume expansion and is then digested into NT-proBNP [9]. Recent studies have revealed that BNP and NT-proBNP may also play a role in predicting stroke in patients with AF. One possible reason for this is that increased pressure on atrial myocytes can lead to increased BNP secretion, thus reflecting atrial dysfunction [10]. Natriuretic peptides (NPs) have been shown to be elevated in AF, with higher levels of BNP and NT-proBNP being predictive of incidental AF [11]. Although no significant difference between NT-proBNP and BNP for HF has been identified in terms of diagnostic efficacy, prognostic evaluation, or outcome time [12], the association between the AF progression phenotype and NT-proBNP is related to the type of AF. In different types of AF, NT-proBNP has exhibited different sensitivities to AF progression [10]. However, relevant studies remain scarce.

This study aims to investigate the correlation between BNP and thromboembolic and atherosclerotic events in patients with NVAF to improve the management of these patients.

## 2. Data and Methods

### 2.1. Study Participants

This is a prospective cohort study. A total of 180 patients with NVAF who underwent pulmonary vein isolation-based radiofrequency ablation at Chongming Hospital, affiliated with Shanghai Health Medical College, were enrolled between June 2015 and March 2022. None of the patients had previously received long-term anticoagulant therapy before admission: they were newly diagnosed with NVAF and a few patients discontinued anticoagulants on their own because they had adapted to the symptoms of AF and had low HAS-BLED and CHA2DS2-VASc scores. For all patients, BNP levels were tested for admission and regular follow-up was performed.

The patients were divided into three groups based on a previous study and the tertiles of the BNP levels of all patients at admission [13]: group A had a BNP of <334.5 pg/mL (low BNP group), group B had a BNP of 334.5–1,288 pg/mL (slightly elevated BNP group), and group C had a BNP of >1,288 pg/mL (significantly elevated BNP group).

### 2.2. Inclusion and Exclusion Criteria

The inclusion criteria were as follows: 65–85 years old; a diagnosis of NVAF confirmed through electrocardiography or a Holter electrocardiogram (NVAF was defined as a type of AF without mitral stenosis or prosthetic valve, no history of cardiac surgery, and the absence of severe mitral regurgitation (≥grade 3 according to colour Doppler echocardiography)) [14, 15]; and no previous anticoagulant therapy before admission. The exclusion criteria were as follows: patients with AF caused by hyperthyroidism; severe systemic disease requiring hospitalisation (e.g., significant liver disease, overt organ failure, neurologic disorders, or malignant disease), significant valvular heart disease, severe infection, acute pulmonary oedema, and acute myocardial infarction (MI); history of cardiac surgery; uncontrolled hypertension and diabetes; and patients who were unable to cooperate with the study. This study was approved by the Ethics Committee of Chongming Hospital, affiliated with Shanghai Health Medical College, and all patients provided informed consent.

### 2.3. Collection of Information

The collected data included the following: general information like age, sex, serum creatinine levels, BNP, smoking history, and blood lipids; history of combined disease such as hypertension, diabetes, hyperlipidaemia, coronary heart disease, and previous stroke history; and CHA2DS2-VASc score (one point each for HF, hypertension, age of 65–74 years, diabetes, vascular disease, and female sex and two points each for age of >75 years and previous history of stroke/transient ischaemic attacks/thromboembolic). The total score was 9 points and constituted the HAS-BLED score.

### 2.4. Follow-Up

All patients received long-term anticoagulation therapy with dabigatran or rivaroxaban after radiofrequency ablation. Follow-up was performed in the outpatient clinic or by telephone after 6 months and 1 year after radiofrequency ablation. The total duration of follow-up was 1 year. Previous literature shows that there is no difference in the probability of cardiac adverse events between patients who receive telephone or online remote follow-ups and patients who receive outpatient clinic follow-ups. This emphasises the feasibility of follow-up without increasing the workload of outpatient clinics [16].

The follow-up items included whether the patient had thromboembolism, atherosclerosis, or adverse events at the time of follow-up. The occurrence of thromboembolic and atherosclerotic events was the primary follow-up endpoint, and they included ischaemic stroke, MI, and systemic embolism. Adverse events were secondary follow-up endpoints, and they included cardiovascular death, all-cause death, and hospitalisation for HF. Patients were also required to provide a hospital-presented diagnostic certificate for the corresponding endpoint event, thereby ensuring the credibility of the follow-up.

### 2.5. Statistical Methods

Statistical analysis of the data was performed using the SPSS 16.0 software. The measurement data of normal distribution were expressed as the mean ± standard deviation. The means of two groups were compared using the *t* test and those of multiple groups using analysis of variance. The statistical description of the nonnormally distributed data was expressed using the mean rank, with an independent samples test used for a comparison between the two groups. The K-independent samples test was used for comparison among multiple groups. A *P* value of <0.05 was considered statistically significant, and the count data were expressed as a rate using the crosstabs test. Items with *P* < 0.05 in univariate comparisons among multiple groups were analyzed using multivariate logistic regression to explore the BNP levels on endpoint events. The test level was *α* = 0.05.

## 3. Results

### 3.1. Group Allocation

A total of 180 patients with NVAF were included in this study, with 110 male patients (61.1%) and 70 female patients (38.9%). Group A (BNP ≤334.5 pg/mL) was the normal BNP group and included 50 patients, group B (BNP = 334.5–1,288 pg/mL) was the slightly elevated BNP group, with 65 patients, and group C (BNP ≥1,288 pg/mL) was the significantly elevated BNP group, with 65 patients. During the 1-year-point follow-up, group A had three primary endpoint events, including three cases of stroke but none of MI or systemic embolization, and five patients had secondary endpoint events. In group B, eight patients had primary endpoint events, including six cases of stroke and two cases of MI. Furthermore, 18 patients had secondary endpoint events. In group C, 17 patients had primary endpoint events, including nine cases of stroke and eight cases of MI. Fifty patients had secondary endpoint events (Table 1).

### 3.2. Baseline Clinical Characteristics

There was no statistical difference in baseline data (age, sex, smoking, coronary artery disease, diabetes, previous stroke, types of AF, SBP, DBP, serum creatinine, HbA1c, total cholesterol, triglycerides, LDL, HDL, and HAS-BLED score) among the three groups (*P* > 0.05).

Group C had the highest CHA2DS2-VASc score, followed by group B and group A, which was statistically significant (*P*=0.001). This indicates that in elderly patients with NVAF, the higher the BNP, the higher the CHA2DS2-VASc score and the greater the thromboembolic risk. Group C had the lowest EF value, followed by group B, with the highest value in group A, and the difference was statistically significant (*P*=0.001). Group C had the largest left atrium (LA) and left ventricular end-diastolic diameter (LVEDD), followed by group B and group A, and the difference was statistically significant (*P*=0.001) (Table 1). Thus, in elderly patients with NVAF, a higher BNP suggests that patients may have lower EF and larger LA and LVEDD, indicating poorer cardiac function.

### 3.3. Occurrence of Endpoint Events

The incidence of primary endpoint events in the three groups was 6.0%, 12.3%, and 26.2%, respectively, and the difference was statistically significant (*P*=0.008). The incidence of adverse events in the three groups was 10.0%, 27.7%, and 76.9%, respectively, and the difference was statistically significant (*P*=0.001) (Table 1). The results indicate that the higher the BNP level, the higher the incidence of stroke and other thromboembolic and atherosclerotic events, as well as other adverse events such as cardiovascular death, all-cause death, and hospitalisation for HF. An elevated BNP suggests an increased risk of complications and poor prognosis in elderly patients with NVAF.

### 3.4. Correlation between Brain Natriuretic Peptide Levels and Thromboembolic and Atherosclerotic Events in Patients with Nonvalvular Atrial Fibrillation

Variables with *P* < 0.05 from the baseline data on admission were included in the multivariate logistic regression analysis, suggesting that elevated BNP was an independent risk factor for thromboembolic and atherosclerotic events in elderly patients with NVAF (OR = 1.001; *P*=0.001). An elevated BNP predicts an increased risk of thromboembolism and atherosclerosis in elderly patients with NVAF (Table 2).

## 4. Discussion

This study revealed that an elevated BNP was associated with thromboembolic and atherosclerotic events in patients with NVAF. The incidence of thromboembolic and atherosclerotic events was higher in the two elevated BNP groups than in the normal BNP group. For stroke or other thromboembolic and atherosclerotic events in patients with an elevated BNP, treatment is also more difficult due to the high BNP and poor cardiac function, often with a catastrophic outcome [17]. Studies have found that patients with HF and AF tend to be elderly and have relatively high NT-proBNP levels [18]. Moreover, AF and HF have common pathophysiological mechanisms and can predict each other independently [19, 20]. One study found that AF increased cardiovascular death and hospitalisation rates for HF [6] and was closely associated with the occurrence and worsening of HF [21]. Furthermore, HF can promote atrial structural changes and electrical remodelling, promoting the occurrence of AF [21].

The NP system is an indicator of myocardial tension, and increased myocardial tension can induce AF [22]. In patients with HF, endothelial dysfunction, hypercoagulability of blood, and blood stasis caused by atrial and ventricular dysfunction contribute to the prothrombotic state, which increases the risk of thrombosis and stroke. This study demonstrated that the incidence of stroke and MI was higher in the elevated BNP group than in the normal BNP group, and the higher the BNP level, the higher the incidence of stroke and MI. Studies have revealed that patients with HF have a high incidence of stroke [23] and that patients with HFrEF, who have a high risk of stroke, may benefit from anticoagulant therapy [24]. The activation of the coagulation system in HF is also a key factor in atrial thrombosis. The combination of HF and AF undoubtedly increases the probability of thrombosis in the atrium, and according to the observation results, the incidence of thromboembolic events is as high as 28% [17, 20]. Therefore, active and sufficient anticoagulation strategies should be adopted for patients with AF and elevated BNP to minimise the occurrence and reduce the disability rate of stroke and MI.

Elderly patients tend to have multiple diseases and often have a relatively high risk of bleeding due to anticoagulation and poor compliance with anticoagulant therapy. Patients aged 65 years and older were mainly selected for this study. Studies have associated HF and a high level of NT-proBNP (>400 pg/mL) with AF after ischaemic stroke [25]. Data indicate that in patients over 65 years with vascular disease, hypertension, diabetes mellitus, and/or HF, the risk of ischaemic stroke is equivalent to a high risk of AF thrombosis [26, 27]. Moreover, elderly patients with AF and HF have a higher incidence of ischaemic stroke. The reason for this may be that the decrease in cardiac output in patients with HF, which leads to a decrease in cerebral blood flow, promotes the occurrence of nonembolic stroke.

Although NP is mainly secreted by ventricular myocytes, Inoue et al. found that in patients with AF, BNP can be secreted by the atrium [28]. Atrial overload and remodelling that occurs in AF are thought to underlie elevated BNP levels. Left ventricular filling pressure caused by the loss of atrial contraction in AF may contribute to an increased left atrial volume, which, in turn, leads to increased BNP levels. Moreover, BNP is an important biomarker of HF and a key indicator of the severity of HF. Patients with AF and HF have a greater probability of ischaemic stroke and a worse prognosis. This study found that the higher the BNP level, the greater the thromboembolic and atherosclerotic risk. Patients with NVAF and HF have a high incidence of ischaemic stroke. Furthermore, multivariate logistic regression analysis revealed that BNP was an independent risk factor for thromboembolic and atherosclerotic events in elderly patients with NVAF. This indicates that BNP can be used as a key predictor of stroke and MI incidence in patients with NVAF and HF. For patients with NVAF and elevated BNP, clinicians should be fully aware of thromboembolism risk and should regulate anticoagulation, actively correct HF, guide patients to follow a reasonable diet and undertake appropriate exercise, and minimise the occurrence of thromboembolism.

This study has some limitations. First, only baseline BNP levels were recorded for the patients admitted. Although this could reflect HF to some extent, the values were not recorded during the follow-up period, preventing the exploration of BNP levels on thromboembolic and atherosclerotic events. Second, only outpatient or telephone follow-ups were conducted, and the frequency of follow-up was low, meaning more detailed and accurate prognostic information could not be provided. Finally, this study is a prospective observational cohort study with a small sample size. Although confounding factors such as age, sex, and previous embolism history have been corrected, unknown and uncorrected residual confounding factors may exist that may have influenced the findings to some extent. The measurement of BNP can be undertaken in either the outpatient clinic or on a ward. The predictive threshold of BNP could enable the improved comprehensive management of patients and further determine the intensity of subsequent anticoagulation therapy, provide preliminary ideas on whether patients with elevated BNP need an intensive anticoagulation regimen, and potentially guide established AF centres in further research.

In conclusion, for patients with NVAF and insignificant HF symptoms, BNP may be related to the risk of thromboembolic and atherosclerotic events, and BNP levels may help guide the formulation of anticoagulation regimens.

## Figures and Tables

**Table 1:** Comparison of baseline data, primary endpoint events, and adverse events among the three groups.

Items	Group A (*n* = 50)	Group B (*n* = 65)	Group C (*n* = 65)	*Z* or *χ*^2^	*P*
Age (mean ± SD)	73.0 ± 4.6	74.1 ± 5.2	74.0 ± 5.0	0.693	0.501
Male (*n* (%))	28 (56.0%)	38 (58.5%)	44 (67.7%)	1.926	0.382
Smoke (*n* (%))	21 (42.0%)	27 (41.5%)	33 (50.8%)	1.371	0.504
DM (*n* (%))	17 (34.0%)	23 (35.4%)	29 (44.6%)	1.721	0.423
CAD (*n* (%))	13 (26.0%)	17 (26.2%)	26 (40.0%)	3.751	0.153
Previous stroke (*n* (%))	11 (22.0%)	9 (13.8%)	14 (21.5%)	1.693	0.429

*Types of AF*					
Paroxysmal AF (*n* (%))	43 (86.0%)	55 (84.6%)	53 (81.5%)	1.753	0.357
Persistent AF (*n* (%))	7 (14.0%)	10 (15.4%)	12 (18.5%)		
SBP (mmHg)	123.5 ± 14.1	119.3 ± 15.6	121.5 ± 14.4	1.152	0.318
DBP (mmHg)	69.0 ± 8.7	70.2 ± 11.0	71.4 ± 10.5	0.344	0.709
BNP (mean ± SD)	120.8 ± 83.3	801.1 ± 276.2	2256.1 ± 861.1	118.462	0.001
Cr (mean ± SD)	72.4 ± 15.3	77.5 ± 16.9	80.3 ± 40.8	1.167	0.314
HbA1c (mean ± SD)	6.4 ± 1.2	6.3 ± 1.4	6.5 ± 1.5	0.352	0.704
TC (mean ± SD)	4.3 ± 1.1	4.4 ± 1.0	4.1 ± 1.0	0.868	0.422
TG (mean ± SD)	1.7 ± 1.0	1.5 ± 0.6	1.7 ± 1.2	1.183	0.309
HDL (mean ± SD)	1.1 ± 0.2	1.1 ± 0.2	1.1 ± 0.3	0.051	0.950
LDL (mean ± SD)	2.4 ± 0.8	2.5 ± 0.8	2.3 ± 0.8	0.817	0.443
CHA2DS2-VASc (mean ± SD)	3.3 ± 1.2	3.5 ± 1.1	4.1 ± 1.1	15.142	0.001
HAS-BLED (mean ± SD)	2.1 ± 0.6	2.0 ± 0.5	2.1 ± 0.5	3.575	0.167
EF (mean ± SD)	63.6 ± 5.6	46.7 ± 7.3	39.0 ± 4.6	119.893	0.001
LA (mean ± SD)	28.1 ± 3.0	33.9 ± 4.6	41.3 ± 5.5	105.031	0.001
LVEDD (mean ± SD)	43.84 ± 3.40	46.5 ± 4.0	54.1 ± 6.0	74.430	0.001
Main endpoint event (*n* (%))	3 (6.0%)	8 (12.3%)	17 (26.2%)	9.556	0.008
Adverse event (*n* (%))	5 (10.0%)	18 (27.7%)	50 (76.9%)	59.485	0.001

*Note*. SD, standard deviation; DM, diabetes mellitus; CAD, coronary artery disease; AF: atrial fibrillation; SBP: systolic blood pressure; DBP: diastolic blood pressure; Cr, creatinine; HbA1c, hemoglobin A1c; TC, total cholesterol; TG, triglycerides; HDL, high-density lipoprotein; LDL, low-density lipoprotein cholesterol; EF, ejection fraction; LA, left atrial; LVEDD, left ventricular end-diastolic diameter.

**Table 2:** The independent risk factor for thromboembolic and atherosclerotic events in elderly NVAF patients.

	B	SE	Wald	*P*	OR	95% CI
BNP	0.001	0.000	10.845	0.001	1.001	1.000–1.001
EF	0.014	0.042	0.115	0.735	1.014	0.934–1.102
LVEDD	−0.048	0.073	0.431	0.511	0.953	0.827–1.099
LA	0.014	0.093	0.023	0.880	1.014	0.845–1.218
CHA2DS2-VASc	0.327	0.204	2.560	0.110	1.386	0.929–2.068
Constant	−3.067	3.974	0.596	0.440	0.047	

*Note*. OR, odds ratio; EF, ejection fraction; LA, left atrial; LVEDD, left ventricular end-diastolic diameter.

## Data Availability

All data generated or analyzed during this study are included within this article.
